# Serological Evidence for Influenza A Virus Exposure in Wild Birds in Trinidad & Tobago

**DOI:** 10.3390/vetsci5020050

**Published:** 2018-05-09

**Authors:** Arianne Brown Jordan, Darshan Narang, Steve C. Essen, Sharon M. Brookes, Ian H. Brown, Christopher Oura

**Affiliations:** 1 Department of Basic Veterinary Sciences, School of Veterinary Medicine, The University of the West Indies, St. Augustine, Eric Williams Medical Sciences Complex, Mount Hope, Trinidad and Tobago; arianne.brown@my.uwi.edu; 2 Department of Life Sciences, Faculty of Science and Technology, The University of the West Indies, St. Augustine, Eric Williams Medical Sciences Complex, Mount Hope, Trinidad and Tobago; dsn95@yahoo.com; 3 Department of Virology, Animal and Plant Health Agency (APHA), Weybridge, Addlestone, Surrey KT15 3NB, UK; steve.essen@apha.gsi.gov.uk (S.C.E.); sharon.brookes@apha.gsi.gov.uk (S.M.B.); ian.brown@apha.gsi.gov.uk (I.H.B.)

**Keywords:** wild birds, Trinidad & Tobago, Caribbean, avian influenza virus

## Abstract

Migratory waterfowl and shorebirds are known to be important reservoirs for influenza A viruses (IAV) and they have been repeatedly implicated as causing avian influenza virus (AIV) outbreaks in domestic poultry flocks worldwide. In recent years, wild birds have been implicated in spreading zoonotic H5 influenza viruses to many countries, which has generated high levels of public health concern. Trinidad and Tobago (T&T) is positioned along the wintering route of migratory birds from the Americas; every year, many species of wild birds stopover on the islands of T&T, potentially carrying AIVs and exposing local populations of wild and domestic birds, including commercial poultry, to infection. The aim of this study was to trap, sample, and test as many wild bird species as possible to see whether they were actively infected or previously exposed to AIV. A total of 38 wild birds were trapped, sampled, and tested for IAV RNA, antibodies specific for influenza A nucleoprotein (NP) and antibodies that were specific for H5 and H7 subtypes. Five of the samples tested antibody positive for IAV, while three of these samples had positive titres (≥16) for the H5 subtype, indicating that they were likely to have been previously infected with an H5 IAV subtype. One of the samples tested positive for IAV (M gene) RNA. These results highlight the potential threat that is posed by wild birds to backyard and commercial poultry in T&T and emphasise the importance of maintaining high levels of biosecurity on poultry farms, ensuring that domestic and wild birds are not in direct or indirect contact. The results also underline the need to carry out routine surveillance for AIV in domestic and wild birds in T&T and the wider Caribbean region.

## 1. Introduction

Trinidad and Tobago (T&T) consists of two main larger islands that are mostly made up of plains, hills, and low mountains. There are 21 additional smaller islands that are dotted around the coastal areas of both main islands. The geographical terrain of T&T is vast and unique in that it is home to different terrestrial ecosystems that are shaped by the climate, waters, soil, and landforms [1,2]. Distinctively, the wetland areas are some of the largest in the Caribbean, and as such, the country is a unique transit point for birds migrating from both North and South America. T&T is one of the richest locations worldwide for wild birds [3] and is within the top 10 locations worldwide for the number of species of birds per square mile [4]. Charadriiformes (gulls and shorebird species), Procellariiformes (shearwaters), and Anseriformes (waterfowl species) inhabit or pass through T&T during winter months [3,5] and many of these species are known to be potential reservoirs of Influenza A virus (IAV) and have been previously implicated in disease spread [6]. 

IAV is an Orthomyxovirus, with differing subtypes based on the 18 Hemagglutinin and 11 Neuraminidase surface glycoproteins [7]. Subtypes exist of varying pathogenicity, from highly pathogenic to low pathogenicity, across bird species, especially poultry [8,9]. Avian influenza virus (AIV) can cause severe respiratory clinical signs in susceptible birds and can result in high levels of morbidity and mortality in populations [8]. Outbreaks can be extremely difficult to control and the economic impact from losses can be extensive, thus impacting local food security. The 2015 outbreak of highly-pathogenic HPAIV in the United States of America (USA), which originated in wild birds, resulted in the direct losses of over US $1.6 billion [10] for the US poultry industry. This large outbreak was the first time that the Eurasian Goose/Guangdong lineage of H5 HPAIV had been reported in the Americas, representing the further transcontinental spread of these viruses [11]. Wild birds are often as considered ‘reservoirs’ of AIV, as they may not display clinical signs of disease, despite being infected with the virus [12]. Problems arise when AIV is transmitted from wild to domestic birds, resulting in severe and very costly outbreaks [13]. 

There are currently no published accounts that are related to AIV in wild birds in T&T. Two recent serological studies in layer and backyard poultry in T&T revealed no evidence for antibodies to AIV in the sampled birds [14,15]. In Barbados, one of T&T’s nearest island neighbours, AIV (H4N3) was isolated from samples taken from waterfowl (Anseriformes) [16]. In South America, low pathogenicity (LP) strains of AIV of the H7N3 were detected in wild birds in Bolivia and H7N3 (2002), H13N9, H13N2, H5N9 (2007–2009) were detected in wild birds from Chile [17,18]. Interestingly, Chile experienced a HPAIV outbreak in commercial poultry flocks in 2002, which prompted the establishment of continued wild bird surveillance [17]. 

The regular movement and the migration of wild birds within and across the Americas and Caribbean regions poses considerable risk for the spread of AIV to domestic poultry in T&T and the wider Caribbean region, particularly since the current Eurasian lineage of H5 HPAIV is now proven to be able to spread via wild birds to the Americas. The aim of this study was therefore to investigate the infection status of seabirds, shorebirds, and waterfowl species for AIV, and to assess the hazard and risk that is posed by wild birds to domestic poultry species with respect to interspecies transmission. 

## 2. Materials and Methods

### 2.1. Sampling Sites and Sampling Strategy

**Figure 1:**
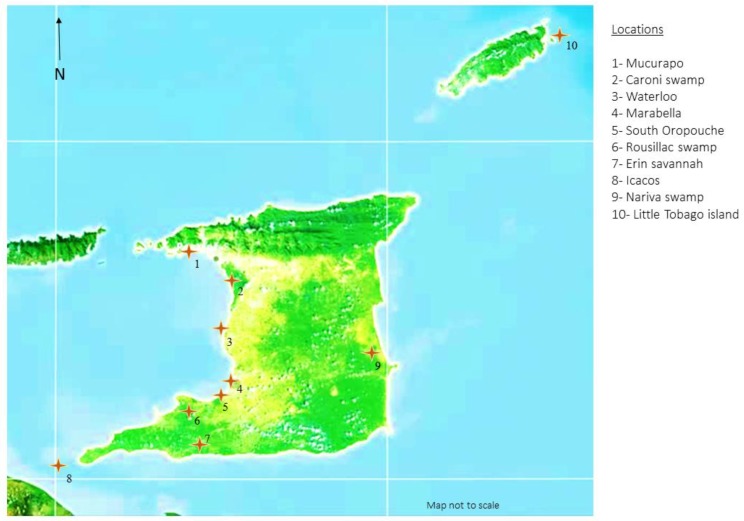
Identified wetland and coastal sampling sites in Trinidad and Tobago.

No fixed sample size was established for this study, as sampling was targeted and convenience based. Sample sites for trapping and sampling of waterfowl, seabirds, and shorebirds were identified from wetland and breeding ground locations across T&T. Locations were chosen based on vehicular accessibility, footpath accessibility, land topology (flat ground), density of avian species habitation, and remoteness (Figure 1). A whoosh net trapping system was utilised to capture birds at foraging/roosting time, while some breeding birds were hand-captured while nesting. Oropharyngeal swabs, cloacal swabs, and blood samples were obtained where possible from the captured birds. All of the birds were clinically examined at the time of sampling and no clinical signs of respiratory disease or other signs of ill health were observed in any of the birds.

### 2.2. Antibody Testing

Serum samples were tested for the presence of anti-nucleoprotein (NP) antibodies to IAV by enzyme-linked immunosorbent assay (ELISA) using the ID Screen^®^ Influenza A Antibody Competition multi-species ELISA kit (ID.Vet, Grabels, France), following the manufacturer’s instructions. Samples that tested positive for IAV antibodies by ELISA were further tested by a Haemagglutination Inhibition (HI, as per the OIE (World Organisation for Animal Health) manual [19]) test at the Animal and Plant Health Agency (APHA), Weybridge, UK. Antibody titres in the serum samples were measured against the following AIV strains: A/chicken/Scotland/59 H5N1 (HPAIV), A/teal/England/7394-2805/06 H5N3 (LPAIV), A/duck/England/036254/14, H5N8 (HPAIV), and A/turkey/England/647/77 H7N7 (LPAIV).

### 2.3. RNA Extraction Real-Time Reverse Transcription Polymerase Chain Reaction (qRT-PCR)

RNA was extracted from all of the swab samples using the Maxwell 16 Viral Total Nucleic Acid Purification kit (Promega, WI, USA) and the Maxwell 16 instrument (Promega, WI, USA). Real-time reverse transcription polymerase chain reaction (qRT-PCR) was carried out on the extracted RNA following a previously published protocol for IAV—pan-influenza A (M gene) assay [18]. All of the positive samples were re-extracted in duplicate and the qRT-PCR was repeated in duplicate.

### 2.4. Ethical Clearance

Ethical approval and relevant licences allowing the wild bird sampling were obtained from The University of the West Indies (St. Augustine), the Ministry of Agriculture, Land and Fisheries Wildlife Division and the Tobago House of Assembly, Division of Agriculture, Marine Affairs, Marketing and Environment.

## 3. Results

A total of 38 wild birds were trapped, sampled, and tested for IAV RNA, antibodies specific for IAV (NP) and antibodies specific for a sub-set (H5 & H7) of IAV subtypes (see Table 1). All of the birds were in good health at the time of sampling and no clinical signs were observed. Five (5/16, 31%) of the samples tested antibody positive for IAV by ELISA. All the other samples tested antibody negative by ELISA. Three of the ELISA positive samples (3/5) were observed to have significant titres of 64, 64, and 512 against the H5 subtype (H5N3) through HI testing according to international standards. No reactivity was observed with the H7 antigen, nor H5 antigens that were derived from the contemporary Goose/Guangdong HPAI virus lineage. One of the samples tested positive for IAV RNA by qRT-PCR (Table 1). 

## 4. Discussion

The identification of high titres of antibodies against a low pathogenicity H5N3 AIV (A/teal/England/7394-2805/06 H5N3) in three wild birds in T&T indicates that these birds had been previously infected with an H5 AIV. The H5 antibodies that were detected were most likely induced following infection with a low pathogenicity H5 AIV strain based on the characteristics of the antigens used in the positive HI tests. The possibility that these birds had survived an infection with a highly pathogenic H5 subtype cannot be excluded, but is considered less likely owing to negative results with antigens that were derived from contemporary strains of transboundary H5 viruses whose spread is known to be mediated by wild birds. The evidence for prior exposure to AIV in wild birds in T&T is not surprising, given that wild birds are known reservoirs of these viruses. Even though it is most likely that the sampled birds had been exposed to a LPAIV, there is always a risk that low pathogenicity H5 viruses may mutate to highly pathogenic strains, especially if they are circulating in a non-naïve poultry population. Wild migratory birds carrying low pathogenicity AIVs may come into contact with birds carrying highly pathogenic AIVs, resulting in possible reassortment between the two viruses. Interestingly, IAV RNA was also detected by qRT-PCR in one of the birds that was antibody positive with the pan-influenza ELISA, although no significant antibody titre was detected for this sample in the HI assays for H5 and H7 (Table 1). The reason for this could be that the AIV strain circulating in this bird was not an H5 or an H7 strain, as antibodies were not detected against the H5 and H7 subtypes that were used in the HI test, but antibodies were detected by the generic IAV ELISA. It is therefore possible that antibodies against another subtype of AIV were being detected in this bird. This could have also been the case for sample 5, where no antibody titre was observed against the four H5 and H7 subtypes used in the HI test, but the sample was ELISA positive (Table 1).

The positive identification of AIV antibodies and RNA in wild bird samples, highlights the heightened risk of transmission of AIV from wild birds to domestic poultry species, resulting in the possible outbreaks of disease. This observation emphasises the need for continued monitoring and surveillance, especially when outbreaks of AIV occur in countries like USA and Mexico, which are on the wild bird migratory pathways linking North, Central, South America and the Caribbean. If a low pathogenicity H5 AIV was identified in domestic poultry in T&T, it would be considered to be an OIE notifiable disease, and, according to OIE standards, some intervention action would be needed. How a country would approach controlling a low pathogenicity AIV outbreak in domestic poultry is however purely a country level decision. 

There are a number of factors that contribute to the potential spread of AIV from wild bird populations, to poultry. Physical environmental factors, such as land topography, shoreline gradient, surface water availability, and climate, as well as anthropogenic environmental factors, such as population density, poultry farm type, livestock density, and presence of roads, play a role in increased risk. T&T has many unique sites attracting wild birds across its terrestrial landscape and along coastal areas, marshlands, and inland watershed areas. Well over one hundred species of wild birds are known to migrate to T&T from wintering countries [20]. Species, such as ruddy turnstones, blue-winged teals, and Eurasian pigeons have been recorded in T&T [3]. These and other species are known to be potential reservoirs for AIV [16,21,22]. 

In T&T, there are many opportunities for transmission of AIV between migratory birds, resident wild birds, and domestic/commercial poultry populations. In certain coastal areas, shorebirds share the same sites as resident wild bird species, as well as migratory species such as gulls and terns. Many backyard poultry farms in T&T are open plan and few measures are put in place to prevent wild birds from visiting farms and interacting closely with domestic poultry. Additionally, some backyard farms are located in wetland areas, close to lakes and ponds that wild birds frequently visit. Indeed, there are some poultry farms in T&T where various species of wild and domestic poultry actively share the same terrain, food, and water.

## 5. Conclusions

In this study, positive antibody titres to an H5 AIV were detected in samples from three wild birds, and influenza A viral RNA was detected in a swab sample from one IAV antibody positive bird. These results emphasise the need to maintain regular surveillance for AIV in both wild and domestic birds, as well as to maintain high levels of biosecurity on poultry farms, in order to avoid contact between wild birds and domestic poultry. More detailed mapping of wild bird migratory pathways are required, so risk factors can be better assessed. Additionally, this work emphasises the need for greater levels of collaboration between public health, veterinary health, and environmental and wildlife divisions, so more efficient surveillance and prevention strategies can be implemented in the country and across the region.

## Figures and Tables

**Table 1:** Real-Time Reverse Transcription Polymerase Chain Reaction (qRT-PCR), enzyme-linked immunosorbent assay (ELISA), and haemagglutination inhibition (HI) titre results for five wild birds that tested antibody positive for avian influenza by ELISA.

Sample Number	ELISA	AIV PCR	AIV Reference Strains			
			A/Chicken/Scotland/59 H5N1	A/Teal/England/7394-2805/06 H5N3	A/Duck/England/036254/14 H5N8	A/Turkey/England/647/77 H7N7
1	+ve	−ve	4	512	16	<2
2	+ve	+ve	<2	<2	2	<2
3	+ve	−ve	16	64	2	<2
4	+ve	−ve	16	64	4	<2
5	+ve	−ve	<2	<2	<2	<2
